# Editorial: Community series in new insights into innate immune cell-based immunotherapies in cancer, volume II

**DOI:** 10.3389/fimmu.2026.1898947

**Published:** 2026-06-19

**Authors:** Chiara Porta, Mary Poupot-Marsan, Virginie Lafont

**Affiliations:** 1 Department of Pharmaceutical Sciences, University of Eastern Piedmont, Novara, Italy; 2 Center for Translational Research on Autoimmune & Allergic Diseases (CAAD), University of Eastern Piedmont, Novara, Italy; 3 Centre de Recherche en Cancérologie de Toulouse (CRCT), UMR1037 Institut National de la Santé et de la Recherche Médicale (Inserm)-Univ. Toulouse III Paul Sabatier-ERL5294 Centre National de la Recherche Scientique (CNRS), Oncopole de Toulouse, Toulouse, France; 4 Institut de Recherche en Cancérologie de Montpellier (IRCM), Institut National de la Santé et de la Recherche Médicale (INSERM) U1194, Univ Montpellier, Institut du Cancer de Montpellier (ICM), Montpellier, France

**Keywords:** CAR-NK, Erk5, innate immunity, metabolic reprogramming, NK cells, trained immunity, transcriptomics, tumor microenvironment (TME)

## Introduction

Adaptive T cell-centric immunotherapies, such as immune checkpoint inhibitors (ICIs) and Chimeric Antigen Receptor (CAR) T-cell therapies, have fundamentally transformed clinical oncology. However, the limitations of their therapeutic potential are becoming more evident. Many patients fail to achieve durable responses due to tumor-intrinsic escape mechanisms. These challenges include low tumor mutational burden (TMB), loss of human leukocyte antigen (HLA) expression, and an aggressively immunosuppressive tumor microenvironment (TME) that actively excludes or exhausts cytotoxic T cells. To overcome these barriers, the field of immuno-oncology is experiencing a paradigm shift toward harnessing the innate immune system. Innate immune cells, including natural killer (NK) cells, macrophages, dendritic cells, γδ T cells, and other innate-like lineages, possess unique advantages. These cells feature rapid activation kinetics, HLA-independent tumor recognition, high functional plasticity, and a natural capacity to remodel the suppressive TME, thereby orchestrating and rescuing adaptive anti-tumor immunity. Building on the success of the first volume, this Research Topic, **“***Community series in new insights into innate immune cell-based immunotherapies in cancer, volume II***”**, presents a curated collection of five landmark articles. These contributions span basic, translational, and computational immunology, collectively bridging bench discoveries to next-generation clinical designs.

## Overcoming technical and scalability bottlenecks in NK cell engineering

A major hurdle in translating cell-based immunotherapies lies in the scalable manufacturing and genetic engineering of primary immune cells. Standard viral transduction protocols often suffer from low efficiency in NK cells, prompting the search for non-viral alternatives. Addressing the theme of innate cell engineering, Wang et al. establish a highly optimized, nucleofection-based screening platform for CAR candidates in human primary NK cells. By systematically refining transfection parameters and evaluating electroporation conditions, their methodology allows rapid, high-efficiency evaluation of CAR expression and cytotoxicity without the prolonged timelines and safety concerns associated with viral vectors. This protocol provides an agile, bench-to-bedside toolkit to accelerate the preclinical screening of novel CAR-NK constructs.

Parallel to cell engineering is the requirement for robust cellular expansion. Adoptive NK-cell therapies frequently require large numbers of functional, non-exhausted effector cells. Giordano et al. address this clinical bottleneck by demonstrating that a CD94-driven *in vitro* expansion protocol can successfully generate highly functional adaptive NKG2C^+^NKG2A^-^CD57^+^ NK cells from human cytomegalovirus-seropositive (CMV^+^) healthy donors. This specific subset displays superior longevity, enhanced cytokine production, and elevated direct cytotoxicity against leukemia and colorectal cancer cells. This work validates an accessible, cost-effective strategy to harvest and amplify high-potency “off-the-shelf” immunotherapeutic products from readily available donor pools.

## Epigenetic and metabolic rewiring of innate immunity

The traditional view of innate immunity as lacking memory has been dismantled by the discovery of “trained immunity”, epigenetic and metabolic reprogramming of myeloid and NK cells resulting in long-term, non-specific hyper-responsiveness. In a comprehensive review, Qu et al. examine the biological mechanics and clinical significance of trained immunity in oncology. The authors delineate how trained immune stimulants, such as microbial components (e.g., β-glucans, BCG), can reprogram progenitor cells in the bone marrow to generate long-lasting, tumor-resilient myeloid and lymphoid effectors. They discuss how exploiting this innate immunological memory can complement conventional immune checkpoint blockades and alleviate systemic immunosuppression.

In addition to immunological memory, metabolic cross-talk within the TME serves as a critical axis regulating cell function. Tumor cells dynamically rewire their metabolic pathways to survive, which directly impacts surrounding immune cells. Villalba et al. explore this metabolic-signaling cross-talk, highlighting Mitogen-Activated Protein Kinase ERK5 (MAPK ERK5) as a vital nexus. The authors evaluate how tumor-intrinsic fatty acid oxidation (FAO) dictates metastatic potential while simultaneously modulating the activation of ERK5 signaling networks. This pathway fundamentally acts as a molecular “rheostat” that shapes cancer cell susceptibility to NK-mediated immunosurveilllance. Targeting the FAO-ERK5 axis thus represents a promising therapeutic avenue to sensitize resistant tumors to NK-cell effector functions.

## High-resolution microenvironmental deconvolution across disease stages

Designing personalized immunotherapies requires an intimate, spatiotemporal understanding of how the TME changes as a tumor progresses. To profile these dynamics, Hurtado et al. present an advanced computational immunology pipeline, executing transcriptomic profiling of the non-small cell lung cancer (NSCLC) microenvironment across early to advanced disease stages. Crucially, to provide necessary functional validation beyond public database analysis, the authors validate their deconvolution results using independent patient cohorts and single-cell RNA-sequencing (scRNA-seq) datasets. Their analysis uncovers “dual immune cell behaviors,” revealing specific transcription factor networks that drive innate cells, particularly NK subsets, to transition from active anti-tumor effectors in early-stage disease to exhausted or suppressive states as malignancy progresses. These findings provide a framework for timing innate immunotherapies relative to disease staging.

## Future perspectives and concluding remarks

The scientific contributions compiled in this Research Topic underscore the high versatility of innate immune cells in cancer therapy. From non-viral CAR-NK engineering and targeted expansion of potent adaptive NK subsets, to the exploitation of trained immunity, metabolic signaling, and longitudinal microenvironmental profiling, these studies significantly advance our understanding of innate anti-tumor immunity. As the field progresses, successful clinical translation will depend on overcoming persistent challenges, including limited *in vivo* persistence, insufficient tumor homing, and resistance to TME-induced immunosuppression. Additional efforts will also be required to improve the durability, scalability, and standardization of innate immune cell-based therapies, particularly in the context of allogeneic “off-the-shelf” platforms.

Integrating innovative cell-engineering approaches with strategies aimed at modulating the tumor microenvironment will likely be essential for the development of more effective therapies. Importantly, future therapeutic strategies will probably rely on rational combinatorial approaches integrating innate immune cells with immune checkpoint inhibitors, targeted therapies, radiotherapy, or conventional chemotherapy in order to maximize anti-tumor efficacy and overcome resistance mechanisms.

Recent advances in single-cell and spatial multi-omics technologies are also expected to refine our understanding of innate immune cell heterogeneity, functional states, and cellular interactions within the TME. These approaches may facilitate the identification of predictive biomarkers and support the development of personalized immunotherapeutic interventions tailored to individual tumor ecosystems. Moreover, the increasing integration of computational modeling and artificial intelligence into immuno-oncology research may accelerate patient stratification, therapeutic target discovery, and treatment optimization.

We extend our sincere gratitude to all authors, reviewers, and editors whose dedicated efforts made this collection possible. We hope these insights inspire continued discovery and foster multi-target, highly resilient therapeutic approaches that improve clinical outcomes for cancer patients globally.

